# Polypharmacy in multimorbid older adults: protocol for a systematic review

**DOI:** 10.1186/s13643-017-0492-9

**Published:** 2017-05-19

**Authors:** Caroline Sirois, Marie-Laure Laroche, Line Guénette, Edeltraut Kröger, Dan Cooper, Valérie Émond

**Affiliations:** 1 Department of Social and Preventive Medicine, Faculty of Medicine, Laval University, Québec, Canada; 2 Centre d’excellence sur le vieillissement de Québec, Centre de recherche du CHU de Québec, Québec, Canada; 3 Institut national de santé publique du Québec, Québec, Canada; 4 Santé des populations et pratiques optimales en santé, Centre de recherche du CHU de Québec, Québec, Canada; 5 Service de Pharmacologie, Toxicologie et Pharmacovigilance, Centre Régional de Pharmacovigilance, de Pharmacoépidémiologie et d’information sur les médicaments, Centre Hospitalier Universitaire de Limoges, Limoges, France; 6 Faculté de Médecine, Université de Limoges, Limoges, France; 7 Faculté de pharmacie, Université Laval, Québec, Canada; 8 Centre d’excellence sur le vieillissement de Québec, Hôpital du St-Sacrement, 1050 Chemin Ste-Foy, Local L2-28, Québec, (Qc) G1S 4L8 Canada

**Keywords:** Older adults, Polypharmacy, Multimorbidity, Prescription drugs, Chronic diseases, Surveillance

## Abstract

**Background:**

Polypharmacy, the concurrent use of multiple medications, consistently evokes a negative connotation, notably because it is associated with a plethora of adverse events. Nonetheless, the number of individuals exposed to polypharmacy is increasing steeply, especially for older people with multiple diseases. There is a need to carefully study the phenomenon at the population scale to full assess the associated health outcomes. Yet, this reveals a complex task because there exists no consensus indicator of polypharmacy. In fact, the definitions of polypharmacy are heterogeneous and its predisposing factors and associated outcomes are not well defined. The goal of this systematic review is to summarize the literature on polypharmacy in multimorbid individuals aged 65 years and over, targeting three objectives: (1) to identify the definitions of polypharmacy that are used in the context of multimorbidity among older individuals (≥65 years); (2) to ascertain predisposing and concurrent factors associated with polypharmacy; and (3) to describe positive and negative outcomes of polypharmacy among older individuals, including hospitalizations, mortality and costs.

**Methods:**

We will include publications from 2004 to 2016 that target four concepts: polypharmacy, older individuals, multimorbidity and positive/negative outcomes. The search will be performed using EBM Reviews, Embase, Global Health, MEDLINE, AgeLine, CINAHL, Health Policy Reference Center, Public Affairs Index, SocINDEX and Google Scholar. Two independent reviewers will screen the articles, extract the information and evaluate the methodological quality of included studies. The results will be presented in tables and narrative summaries will be performed. We will perform meta-analyses (objective 3) if the heterogeneity is not important.

**Discussion:**

This review will help describe the various ways of conceptualizing polypharmacy and how it is associated with health outcomes. We have selected outcomes most relevant for public surveillance performed with administrative databases. Other positive and negative outcomes have been associated with polypharmacy but may not be included in the review.

**Systematic review registration:**

PROSPERO CRD42014014989

**Electronic supplementary material:**

The online version of this article (doi:10.1186/s13643-017-0492-9) contains supplementary material, which is available to authorized users.

## Background

The number of older individuals exposed to multiple medications has been increasing tremendously in the recent years. In Canada, two thirds of individuals aged 65 years and over use at least 5 medications per year, and 27% uses at least ten [1]. In Ireland, the proportion of older individuals exposed to ≥10 medications has risen from 1.5% in 1997 to 21.9% in 2012 [2]. This increase in medication use is partly explained by the fact that clinical guidelines frequently encourage the use of multiple medications to treat single chronic diseases. However, the impact of using many medications together for different chronic diseases has not been assessed in randomized controlled trials, under controlled environments. The evidence is thus limited to observational data, which nonetheless appear as an optimal design to study complex situations like polypharmacy that are hardly suitable for evaluation in randomized trials. However, observational data are prone to bias and caution must be paid when interpreting results. An increased number of medications has been associated with adverse events in observational studies [3], such as hospitalization, mortality, falls [4], inappropriate prescribing [5], side effects [6] or drug-drug or drug-disease interactions [7, 8]. The benefits of polypharmacy are well less known. Therefore, the decision of adding yet another medication in older patients sometimes becomes a difficult one for clinicians [9, 10].

Obtaining a clear and complete portrait of polypharmacy and its impacts reveals a daunting task. Part of the problem emerges from the fact that there is no consensus on the definition of polypharmacy. Indeed, the literature presents an impressive array of heterogeneous definitions. One approach advocates a definition based on the number of medications, but there is no theoretical basis that may confirm the number of medications required for such a definition [11]. Another approach promotes a definition involving the quality of prescribing, but distinguishing appropriate and inappropriate polypharmacy remains difficult [3]. In fact, defining polypharmacy is a conceptual challenge. The problematic involves complex situations that impose reflections. For example, the pertinence of polypharmacy may vary according to life expectancy, comorbidities or side effects. This aspect has not been addressed thoroughly in systematic reviews conducted until now [12–15].

To fully understand the impacts of polypharmacy, there is a need to identify the predisposing and concurrent factors associated with the use of multiple drugs. Some elements have been associated with polypharmacy, such as older age [12, 16–18], lower education levels [12, 16–18], being a woman [12, 16–18], a recent hospitalization [18] or multiple prescribers [17]. Nonetheless, some inconsistencies have been reported around those factors, which are often not evaluated in relation to other contributing factors. Therefore, the features that favour the development of polypharmacy, the groups that are most susceptible to benefit or suffer from polypharmacy, and the characteristics of polypharmacy (specific medications or combinations, interactions) that are most likely to lead to these outcomes are still not well defined for the older population. Such knowledge is essential to ensure rational decisions in treating older individuals.

When medications are used in accordance with clinical practice guidance, the use of multiple drugs should engender positive impacts in multimorbid patients, but polypharmacy has constantly been associated with adverse outcomes. There is a need to thoroughly search how polypharmacy can also be beneficial, notably in terms of hospitalizations and mortality. Finally, evaluating the costs that are driven by polypharmacy is an important task to establish the direct and indirect impacts that polypharmacy engenders on the health system.

There is a need to gather information about polypharmacy to fully judge its consequences and to develop interventions designed to tackle the issues related to this phenomenon, both at the individual and population level. For example, the *Institut national de santé publique du Québec* (INSPQ) intends to create a population-based surveillance system for polypharmacy, using the Quebec Integrated Chronic Disease Surveillance System (QICDSS) [19]. In order to be useful, the polypharmacy indicators created for surveillance should respond to the needs of all possible knowledge users, including clinicians, researchers, and decision makers. Yet, there is no data on how well the conceptualizations of polypharmacy align among those fields. Exploring definitions and outcomes of polypharmacy will help design indicators that will be relevant for all purposes.

The goal of this systematic review is to summarize the literature on polypharmacy among multimorbid individuals aged 65 years and over. Specifically, we intend the following:

1. To identify the definitions of polypharmacy that are used in the context of multimorbidity among older individuals (≥65 years)
2. To ascertain predisposing (that lead to) and concurrent (that are simultaneously present) factors associated with polypharmacy among older individuals
3. To describe positive and negative outcomes of polypharmacy among older individuals on hospitalizations, mortality and costs.

## Methods

### Participants/population

The review will consider studies that include people aged 65 years and older with at least two concurrent chronic diseases. We will include studies if at least one of the following applies:

- At least 80% of participants are aged 65 years and older.
- The data from people aged 65 years and older can be extracted.

We will include all settings (community, hospital, nursing homes) and types of health care (public, private). We will perform subgroup analyses according to those settings.

### Exposure and comparators

Older individuals with chronic diseases exposed to polypharmacy will be considered. Older individuals with chronic disease not exposed to polypharmacy will be the comparators when applicable (objectives 2 and 3).

### Outcomes

- Objective 1: We will include articles presenting a clear operational definition of polypharmacy.
- Objective 2: We will include studies that quantify the association of predisposing or concurrent factors (e.g. demographic, treatment-related, morbidity, health system-related) with the presence of polypharmacy.
- Objective 3: We will include studies that evaluate the following outcomes of polypharmacy:
  1. Hospitalization or emergency department visits
  2. Mortality
  3. Costs [e.g. direct (medication, hospitalization, medical visits, diagnostic procedures, laboratory procedures) and indirect (e.g. for relatives: productivity loss, early retirement)]

### Searches

A systematic search strategy (see Additional file 1) has been developed by the authors (CS, VE) in collaboration with an experienced librarian (VT). A second librarian specialized in health sciences has revised the proposed strategy (SV). A first search has been performed in December 2014 and was updated in May 2016 using the ovidSP (Bouquet total access collection, EBM Reviews, Embase, Global Health, MEDLINE) and EbscoHost (AgeLine, CINAHL, Health Policy Reference Center, MEDLINE, Public Affairs Index, SocINDEX) platforms. We will also search Google Scholar and Google to identify grey literature, such as governmental reports, reporting on indicators of polypharmacy that have been used for population-based monitoring.

Four concepts have been defined in our search: polypharmacy, older individuals, multimorbidity and positive/negative outcomes. The search strategy has been adapted to the syntax requirements of each database (use of different thesaurus terms, truncation and wildcard characters). We included studies published since 2004 (corresponding to the last 10 years before our first search) in all languages. We will also hand search the bibliographies of all included papers to retrieve studies that have not been identified through the database searches.

### Types of study to be included

There will be no restrictions on the types of study. We will include randomized controlled trials, non-randomized controlled trials, quasi-experimental trials, before and after studies, transversal descriptive studies, surveys, cohort studies, case–control studies, case reports and case series. Reviews, commentaries, editorials and practice guidelines will be used to identify polypharmacy definitions, and they will be searched for the primary references they refer to. Studies evaluating interventions regarding polypharmacy and methodological studies (e.g. those comparing two definitions) will be evaluated. We will also evaluate the grey literature, such as reports, thesis and governmental publications. In case of missing information (both for abstracts and full text), we will contact the authors to complete the required information if the study corresponds to the inclusion criteria. Only those studies published in the last 12 years (2004–2016) will be included to ensure that polypharmacy is representative of today’s pharmacological arsenal and definitions of polypharmacy.

### Study selection

We used Endnote to group the results and exclude duplicated articles. Two independent reviewers will examine the titles and screen the abstracts (MD, CS). Full-text articles will be retrieved for the papers not excluded from the previous two steps. Full text will be reviewed by two independent reviewers (CS and NSD/AZ). Additional information from the study authors will be sought if questions about eligibility arise. In case of disagreement, consensus will be obtained through discussion; if consensus cannot be reached, a third reviewer will be consulted (VE). All exclusion criteria will be recorded at each step (title, abstract and full-text review) in order to create flow charts according to Preferred Reporting Items for Systematic Reviews and Meta-Analyses (PRISMA) requirements (see Additional file 2). The selection process will be repeated independently for the three objectives.

Articles will be excluded on the basis of the following exclusion criteria:

1. The article does not provide an operational definition of polypharmacy. For example, defining polypharmacy as “large number of medications” would not qualify as an operational definition. To be considered operational, the definition can involve a specific number of medications (e.g. 5 medications and more) or indicate a specific condition (e.g. complex medication regimen with at least one inappropriate medication).
2. The studied population do not include people 65 years and over. Studies that include individuals younger than 65 years and over can be included if (a) 80% and more of the study population is 65 years old and (b) there are stratified analyses for the age group 65 years and over that allow the extraction of specific data for this age group.
3. The article refers to polypharmacy used for the treatment of a single medical condition in the absence of multimorbidity. Since the review focuses on individuals with multimorbidity, studies that evaluate a specific disease will be included only if individuals present other concomitant conditions. We will therefore not examine studies that focus on polypharmacy as treatment for a single disease (e.g. polypharmacy for psychiatric conditions can be described as the use of two psychotropic medications).
4. Missing information. The abstracts will be excluded if required information is not included in the abstract and no further information was available after contacting the corresponding author. The same applies for full-text articles.
5. Systematic review. The primary references included in the systematic reviews will be included in our review if they respond to our inclusion criteria. Systematic reviews can be included in objective 1 if they provide an operational definition of polypharmacy.
6. The article does not provide information about predisposing or concurrent factors related to polypharmacy (objective 2 only).
7. The article does not address outcomes of polypharmacy targeted in our review (objective 3 only).
8. The paper was published before 2004.
9. The article is published in a language that our team is not fluent with (English, French, Spanish, German, Portuguese, Arabic) or for which we do not find adequate resources to translate.
10. Duplicate publications. This exclusion criterion regroups studies that are summary of another published article, duplicate of studies already included or response letter to published articles.

### Data extraction

Two independent reviewers (AZ/NSD, CS) will conduct a full-paper evaluation and data extraction. Established data extraction forms adapted for the three objectives of the review have been created using FileMaker^MD^ (https://www.filemaker.com). This will allow all researchers to have access to the data in real time.

For each study, we will record bibliographic details, type of study, context, participants and the definition of polypharmacy. Duplicate studies (articles related to the same specific study) will be excluded (criterion 10). We will record specific information according to each objective:

- Objective 1: We will extract details about the definition of polypharmacy, the types of medications included in the definition, the way the medications have been counted or what qualitative criteria have been used in order to label a medication regimen as polypharmacy (e.g. inappropriate medication). The methods used to define the quality of polypharmacy will be recorded when present. We will compare the definitions used in the publications according to whether the article relates to areas of clinical practice, research or public health. This will allow us to evaluate whether the concept of polypharmacy is consistent between the three areas, and whether to develop a single indicator of polypharmacy that is pertinent for the three areas is realistic. We will also evaluate whether the definition used in the articles relies on a theoretical framework is the result of a methodological assessment, refers to other published material or seems arbitrary. We will regroup definitions according to their nature (numerical definitions only, quality of prescribing only, mixed definitions).
- Objective 2: We will extract factors that are associated with polypharmacy (prevalence) and those associated with its development (incidence), taking into account the definition of polypharmacy. We will group factors in three categories: patient-related (e.g. sex, age); disease-related (e.g. comorbidity, inappropriate medications); and health system-related (e.g., multiple prescribers, insurance type).
- Objective 3: We will extract information on three outcomes: (1) hospitalization/emergency department visits (all-caused, specific causes and hospitalization length); (2) all-cause and specific mortality; (3) direct costs (related to medications, medical visits, emergency unit visits, hospitalization, diagnostic procedures) and indirect costs.

### Risk of bias (quality) assessment

For objectives 2 and 3, two independent reviewers (AZ and (LG, MLL, EK, DC, ND)) will assess the quality of the studies according to the Scottish Intercollegiate Guidelines Network methodology checklists for controlled trials, cohort studies and case–control studies [20–22]. In case of disagreement between two reviewers that cannot be resolved through discussion, a third reviewer will be consulted to reach consensus (CS).

The studies will be rated according to a nominal scale of risk based on the gathered information (critical, serious, moderate or low risk). The quality assessment data will be presented in the table of the results. All data will be interpreted in light of the risk of bias. Subgroup analysis based on quality will be performed if required.

### Strategy for data synthesis

We will conduct a narrative synthesis of individual studies for the three objectives. We will summarize information on study types, population characteristics, settings and outcomes. We expect that the heterogeneity of the definitions of polypharmacy will preclude the possibility of a meta-analysis of the outcomes stated in objective 3. We will use GRADE criteria to appraise the quality of the evidence.

### Analysis of subgroups or subsets

We will analyze the results according to different subgroups if possible: age groups (e.g. 65–74/75–84/85+; or 75 and above, etc.), sex, comorbidities (patients with diabetes/cardiovascular disorders/pulmonary conditions/chemotherapy…), settings (community, hospital, nursing homes) and type of drug plan (public, private).

## Discussion

This review will summarize the data around polypharmacy in older individuals following the criteria of the PRISMA-P checklist (see Additional file 3). The first objective will help us gather the different definitions of polypharmacy used in the context of multimorbidity among older individuals. This collation will notably help evaluate if conceptualization of polypharmacy diverges between different settings and populations, and whether visions of polypharmacy in clinical practice, research and population surveillance are aligned. Considering the heterogeneity of the definitions and the amount of potential data we will likely gather, we do not intend to evaluate the quality of each definition retrieved. As such, this first stage of the review corresponds to the methodology of a scoping review. We acknowledge that a quality assessment of definitions would provide more insight to the work, but at this stage, we do not intend to clarify what should be the best definition of polypharmacy. The review will also help identify in a systematic way the factors that are associated with polypharmacy, and the characteristics of polypharmacy that are linked with increased or decreased risks of hospitalization, mortality costs outcomes. We are under no illusions with regard to the possibility of performing meta-analysis for the third objective of the review. We expect that the heterogeneous definitions will preclude such analysis. There is also a definite possibility that a significant number of studies that report factors associated with polypharmacy be of low quality (e.g. presenting univariate evaluation of factors only), which will limit the conclusions that we can generate from the review.

There are other known limitations to our protocol regarding the information retrieved from the literature. First, since our overarching goal is to determine how polypharmacy is defined and tied to health outcomes, studies focusing on a number of medications without considering a specific definition of polypharmacy will not be included. However, we believe our search strategy will identify most of these papers, which should not be excluded before the full-text stage. We will therefore be able to evaluate how our exclusion criteria could impact the results, and we will discuss this point in our review. Second, considering that research on polypharmacy has been increasing in recent years, we expect that a large number of abstracts will be retrieved from our search and that the full-length papers related to them will not yet be available. We will strive for ensuring that complete information be available for all eligible studies, notably by contacting authors for all abstracts and full text that do not provide required data. Nonetheless, we anticipate that some authors will not answer our queries, which will limit the inclusion of recent evidence into our review. Finally, we have selected a limited number of outcomes for the third objective. Those outcomes should be the most relevant for population surveillance as they can be tracked in administrative databases. However, other important outcomes for clinical practice, such as adverse events and adherence, will not be addressed.

Our review will include a great number of observational studies. This poses challenges because it entails ensuring the associations observed are not biased (e.g. confounding by multimorbidity). Considerable effort must be deployed to ensure quality of such studies is adequately evaluated [23, 24]; our team of experienced pharmacoepidemiologists should be capable of addressing this challenge.

We believe the results of this review will help optimize polypharmacy. The increased knowledge about polypharmacy will benefit students and clinicians because there is an obvious need to improve education about polypharmacy [25, 26]. By looking at definitions that are tied to outcomes, we will help establish what should a polypharmacy indicator comprise. Validated polypharmacy indicators will be useful for public health, researchers and clinicians. They will allow for surveillance but also to identify individuals at risk of suffering from negative impacts of polypharmacy and to target individuals who are most likely to benefit from polypharmacy under certain circumstances.

## Additional files

Additional file 1: Search strategies. Search strategies performed in MEDLINE and Embase. (DOCX 157 kb)

Additional file 2: PRISMA chart. (DOCX 283 kb)

Additional file 3: PRISMA checklist. (DOCX 31 kb)

## Abbreviations

CINAHL: Cumulative Index to Nursing and Allied Health Literature
Embase: Excerpta Medica dataBASE
GRADE: Grading of Recommendations Assessment, Development and Evaluation
MEDLINE: Medical Literature Analysis and Retrieval System Online
PRISMA: Preferred Reporting Items for Systematic Reviews and Meta-Analyses
