# Supplementary material for: Polypharmacy in multimorbid older adults: protocol for a systematic review
Source: Syst Rev. 2017 May 19;6:104. doi: 10.1186/s13643-017-0492-9 (PMC5438541; doi:10.1186/s13643-017-0492-9)
Supplement: Supplementary file 1 — Search strategies. Search strategies performed in MEDLINE and Embase. (DOCX 157 kb) [file 13643_2017_492_MOESM1_ESM.docx]

Only the strategies used for MEDLINE and EMBASE in Ovid, as well as the one used to search EBSCOhost databases are presented. The strategies for the oter dabases are available on demand.

### SEARCH STRATEGY in MEDLINE by Ovid

| **Nos** | **Search** |
| --- | --- |
| 1 | (polypharmac* or poly-pharmac* or polypharmacotherap* or polypharmaco-therap* or polymedication* or poly-medication* or polymedicine* or poly-medicine* or ((poly-drug or polydrug) adj use*) or multipharmac* or multi-pharmac* or multimedication* or multi-medication* or multimedicine* or multi-medicine* or ((multi-drug or multidrug) adj use*) or comedication* or co-medication* or polypragmas* or poly-pragmas* or overprescri* or over-prescri* or underprescri* or under-prescri* or ((multimedicated or multi-medicated or multimedicamented or multi-medicamented or polymedicated or poly-medicated or polymedicamented or poly-medicamented or multidrugged or multi-drugged or polydrugged or poly-drugged) adj1 (patient* or user*)) or (Beers* adj2 Criteria)).ti,ab. |
| 2 | ((many or multiple or different or "a lot" or excessive or antagonis* or combination* or combined or concurrent* or concomitant or "at the same time" or simultaneous*) adj2 (drug* or medicines or medication* or prescription* or pharmaceutical* or (pharmacological adj (therap* or treatment*)))).ti,ab. |
| 3 | ((inappropriate or wrong) adj2 (((drug* or medicines or medication* or pharmaceutical* or (pharmacological adj (therap* or treatment*))) adj1 (use* or intake or in-take or take* or taking)) or prescrib* or prescription*)).ti,ab. |
| 4 | polypharmacy/ |
| 5 | inappropriate prescribing/ |
| 6 | (aged OR aging OR ageing OR elder* OR ((old OR retired) ADJ2 (people* OR patient* OR inpatient* OR in-patient* OR outpatient* OR out-patient* OR client* OR person* OR individual* OR wom#n OR man OR men OR age)) OR older* OR geriatr* OR gerontolog* OR senior* OR senescen* OR retiree* OR sexagenarian* OR septuagenarian* OR octagenarian* OR nonagenarian* OR centenarian* OR supercentenarian* OR veteran*).ti,ab. |
| 7 | aging/ OR aged/ OR "aged, 80 and over"/ OR "health services for the aged"/ OR "homes for the aged"/ OR geriatrics/ |
| 8 | (comorbid* OR co-morbid* OR multimorbid* OR multi-morbid* OR polymorbid* OR poly-morbid* OR copatholog* OR co-patholog* OR multipatholog* OR multi-patholog* OR polypatholog* OR poly-patholog* OR ((Charlson OR Elixhauser) ADJ2 (index* OR score*)) OR ((coexisting OR co-existing) ADJ2 (disease* OR illness* OR patholog*))).ti,ab. |
| 9 | comorbidity/ |
| 10 | (1 OR 2 OR 3) ADJ10 6 |
| 11 | 10 ADJ10 8 |
| 12 | (4 OR 5) AND 7 |
| 13 | 12 AND 9 |
| 14 | 11 OR 13 |

For the may 2016 update, the following requests were added :

| 15 | (2014122$ OR 2014123$ OR 201501$$ OR 201502$$ OR 201503$$ OR 201504$$ OR 201505$$ OR 201506$$ OR 201507$$ OR 201508$$ OR 201509$$ OR 201510$$ OR 201511$$ OR 201512$$ OR 201601$$ OR 201602$$ OR 201603$$ OR 201604$$ OR 201605$$ OR 201606$$).ed. |
| --- | --- |
| 16 | 14 AND 15 |

### Research strategy in embase by Ovid

| **Nos** | **Search** |
| --- | --- |
| 1 | (polypharmac* or poly-pharmac* or polypharmacotherap* or polypharmaco-therap* or polymedication* or poly-medication* or polymedicine* or poly-medicine* or ((poly-drug or polydrug) adj use*) or multipharmac* or multi-pharmac* or multimedication* or multi-medication* or multimedicine* or multi-medicine* or ((multi-drug or multidrug) adj use*) or comedication* or co-medication* or polypragmas* or poly-pragmas* or overprescri* or over-prescri* or underprescri* or under-prescri* or ((multimedicated or multi-medicated or multimedicamented or multi-medicamented or polymedicated or poly-medicated or polymedicamented or poly-medicamented or multidrugged or multi-drugged or polydrugged or poly-drugged) adj1 (patient* or user*)) or (Beers* adj2 Criteria)).ti,ab. |
| 2 | ((many or multiple or different or "a lot" or excessive or antagonis* or combination* or combined or concurrent* or concomitant or "at the same time" or simultaneous*) adj2 (drug* or medicines or medication* or prescription* or pharmaceutical* or (pharmacological adj (therap* or treatment*)))).ti,ab. |
| 3 | ((inappropriate or wrong) adj2 (((drug* or medicines or medication* or pharmaceutical* or (pharmacological adj (therap* or treatment*))) adj1 (use* or intake or in-take or take* or taking)) or prescrib* or prescription*)).ti,ab. |
| 4 | polypharmacy/ |
| 5 | inappropriate prescribing/ |
| 6 | (aged OR aging OR ageing OR elder* OR ((old OR retired) ADJ2 (people* OR patient* OR inpatient* OR in-patient* OR outpatient* OR out-patient* OR client* OR person* OR individual* OR wom#n OR man OR men OR age)) OR older* OR geriatr* OR gerontolog* OR senior* OR senescen* OR retiree* OR sexagenarian* OR septuagenarian* OR octagenarian* OR nonagenarian* OR centenarian* OR supercentenarian* OR veteran*).ti,ab. |
| 7 | aging/ OR aged/ OR aged hospital patient/ OR frail elderly/ OR very elderly/ OR postmaturity/ OR age/ OR senescence/ OR elderly care/ OR geriatric care/ OR geriatric nursing/ OR "home for the aged"/ OR geriatrics/ |
| 8 | (comorbid* OR co-morbid* OR multimorbid* OR multi-morbid* OR polymorbid* OR poly-morbid* OR copatholog* OR co-patholog* OR multipatholog* OR multi-patholog* OR polypatholog* OR poly-patholog* OR ((Charlson OR Elixhauser) ADJ2 (index* OR score*)) OR ((coexisting OR co-existing) ADJ2 (disease* OR illness* OR patholog*))).ti,ab. |
| 9 | comorbidity/ OR Charlson Comorbidity Index/ OR Elixhauser comorbidity index/ |
| 10 | (1 OR 2 OR 3) ADJ10 6 |
| 11 | 10 ADJ10 8 |
| 12 | (4 OR 5) AND 7 |
| 13 | 12 AND 9 |
| 14 | 11 OR 13 |

For the may 2016 update, the following requests were added:

| 15 | (201450 OR 201451 OR 201452 OR 201501 OR 201502 OR 201503 OR 201504 OR 201505 OR 201506 OR 201507 OR 201508 OR 201509 OR 201510 OR 201511 OR 201512 OR 201513 OR 201514 OR 201515 OR 201516 OR 201517 OR 201518 OR 201519 OR 201520 OR 201521 OR 201522 OR 201523 OR 201524 OR 201525 OR 201526 OR 201527 OR 201528 OR 201529 OR 201530 OR 201531 OR 201532 OR 201533 OR 201534 OR 201535 OR 201536 OR 201537 OR 201538 OR 201539 OR 201540 OR 201541 OR 201542 OR 201543 OR 201544 OR 201545 OR 201546 OR 201547 OR 201548 OR 201549 OR 201550 OR 201551 OR 201552 OR 201601 OR 201602 OR 201603 OR 201604 OR 201605 OR 201606 OR 201607 OR 201608 OR 201609 OR 201610 OR 201611 OR 201612 OR 201613 OR 201614 OR 201615 OR 201616 OR 201617 OR 201618 OR 201619 OR 201620 OR 201621 OR 201622 OR 201623).em. |
| --- | --- |
| 16 | 14 AND 15 |

### research stragegie in ebscohost databases

| **Nos** | **Search** |
| --- | --- |
| S1 | TI (polypharmac* OR poly-pharmac* OR polypharmacotherap* OR polypharmaco-therap* OR polymedication* OR poly-medication* OR polymedicine* OR poly-medicine* OR ((poly-drug OR polydrug) W0 use*) OR multipharmac* OR multi-pharmac* OR multimedication* OR multi-medication* OR multimedicine* OR multi-medicine* OR ((multi-drug OR multidrug) W0 use*) OR comedication* OR co-medication* OR polypragmas* OR poly-pragmas* OR overprescri* OR over-prescri* OR underprescri* OR under-prescri* OR ((multimedicated OR multi-medicated OR multimedicamented OR multi-medicamented OR polymedicated OR poly-medicated OR polymedicamented OR poly-medicamented OR multidrugged OR multi-drugged OR polydrugged OR poly-drugged) N1 (patient* OR user*)) OR (Beers* N2 Criteria)) OR AB (polypharmac* OR poly-pharmac* OR polypharmacotherap* OR polypharmaco-therap* OR polymedication* OR poly-medication* OR polymedicine* OR poly-medicine* OR ((poly-drug OR polydrug) W0 use*) OR multipharmac* OR multi-pharmac* OR multimedication* OR multi-medication* OR multimedicine* OR multi-medicine* OR ((multi-drug OR multidrug) W0 use*) OR comedication* OR co-medication* OR polypragmas* OR poly-pragmas* OR overprescri* OR over-prescri* OR underprescri* OR under-prescri* OR ((multimedicated OR multi-medicated OR multimedicamented OR multi-medicamented OR polymedicated OR poly-medicated OR polymedicamented OR poly-medicamented OR multidrugged OR multi-drugged OR polydrugged OR poly-drugged) N1 (patient* OR user*)) OR (Beers* N2 Criteria)) |
| S2 | TI ((many OR multiple OR different OR "a lot" OR excessive OR antagonis* OR combination* OR combined OR concurrent* OR concomitant OR "at the same time" OR simultaneous*) N2 (drug* OR medicines OR medication* OR prescription* OR pharmaceutical* OR (pharmacological W0 (therap* OR treatment*)))) OR AB ((many OR multiple OR different OR "a lot" OR excessive OR antagonis* OR combination* OR combined OR concurrent* OR concomitant OR "at the same time" OR simultaneous*) N2 (drug* OR medicines OR medication* OR prescription* OR pharmaceutical* OR (pharmacological W0 (therap* OR treatment*)))) |
| S3 | TI ((inappropriate OR wrong) N2 (((drug* OR medicines OR medication* OR pharmaceutical* OR (pharmacological W0 (therap* OR treatment*))) N1 (use* OR intake OR in-take OR take* OR taking)) OR prescrib* OR prescription*)) OR AB ((inappropriate OR wrong) N2 (((drug* OR medicines OR medication* OR pharmaceutical* OR (pharmacological W0 (therap* OR treatment*))) N1 (use* OR intake OR in-take OR take* OR taking)) OR prescrib* OR prescription*)) |
| S4 | TI (aged OR aging OR ageing OR elder* OR ((old OR retired) N2 (people* OR patient* OR inpatient* OR in-patient* OR outpatient* OR out-patient* OR client* OR person* OR individual* OR wom?n OR man OR men OR age)) OR older* OR geriatr* OR gerontolog* OR senior* OR senescen* OR retiree* OR sexagenarian* OR septuagenarian* OR octagenarian* OR nonagenarian* OR centenarian* OR supercentenarian* OR veteran*) OR AB (aged OR aging OR ageing OR elder* OR ((old OR retired) N2 (people* OR patient* OR inpatient* OR in-patient* OR outpatient* OR out-patient* OR client* OR person* OR individual* OR wom?n OR man OR men OR age)) OR older* OR geriatr* OR gerontolog* OR senior* OR senescen* OR retiree* OR sexagenarian* OR septuagenarian* OR octagenarian* OR nonagenarian* OR centenarian* OR supercentenarian* OR veteran*) |
| S5 | TI (comorbid* OR co-morbid* OR multimorbid* OR multi-morbid* OR polymorbid* OR poly-morbid* OR copatholog* OR co-patholog* OR multipatholog* OR multi-patholog* OR polypatholog* OR poly-patholog* OR ((Charlson OR Elixhauser) N2 (index* OR score*)) OR ((coexisting OR co-existing) N2 (disease* OR illness* OR patholog*))) OR AB (comorbid* OR co-morbid* OR multimorbid* OR multi-morbid* OR polymorbid* OR poly-morbid* OR copatholog* OR co-patholog* OR multipatholog* OR multi-patholog* OR polypatholog* OR poly-patholog* OR ((Charlson OR Elixhauser) N2 (index* OR score*)) OR ((coexisting OR co-existing) N2 (disease* OR illness* OR patholog*))) |
| S6 | (S1 OR S2 OR S3) N10 S4 |
| S7 | S6 N10 S5 |

For the may 2016 update, the following requests were added:

| S8 | (EM 20141219- OR ED 20141219-) |
| --- | --- |
| S9 | S7 AND S8 |
