# Supplementary figures and images for: Polypharmacy in multimorbid older adults: protocol for a systematic review
Source: Syst Rev. 2017 May 19;6:104. doi: 10.1186/s13643-017-0492-9 (PMC5438541; doi:10.1186/s13643-017-0492-9)

### PRISMA CHART

A chart will be created for each objective


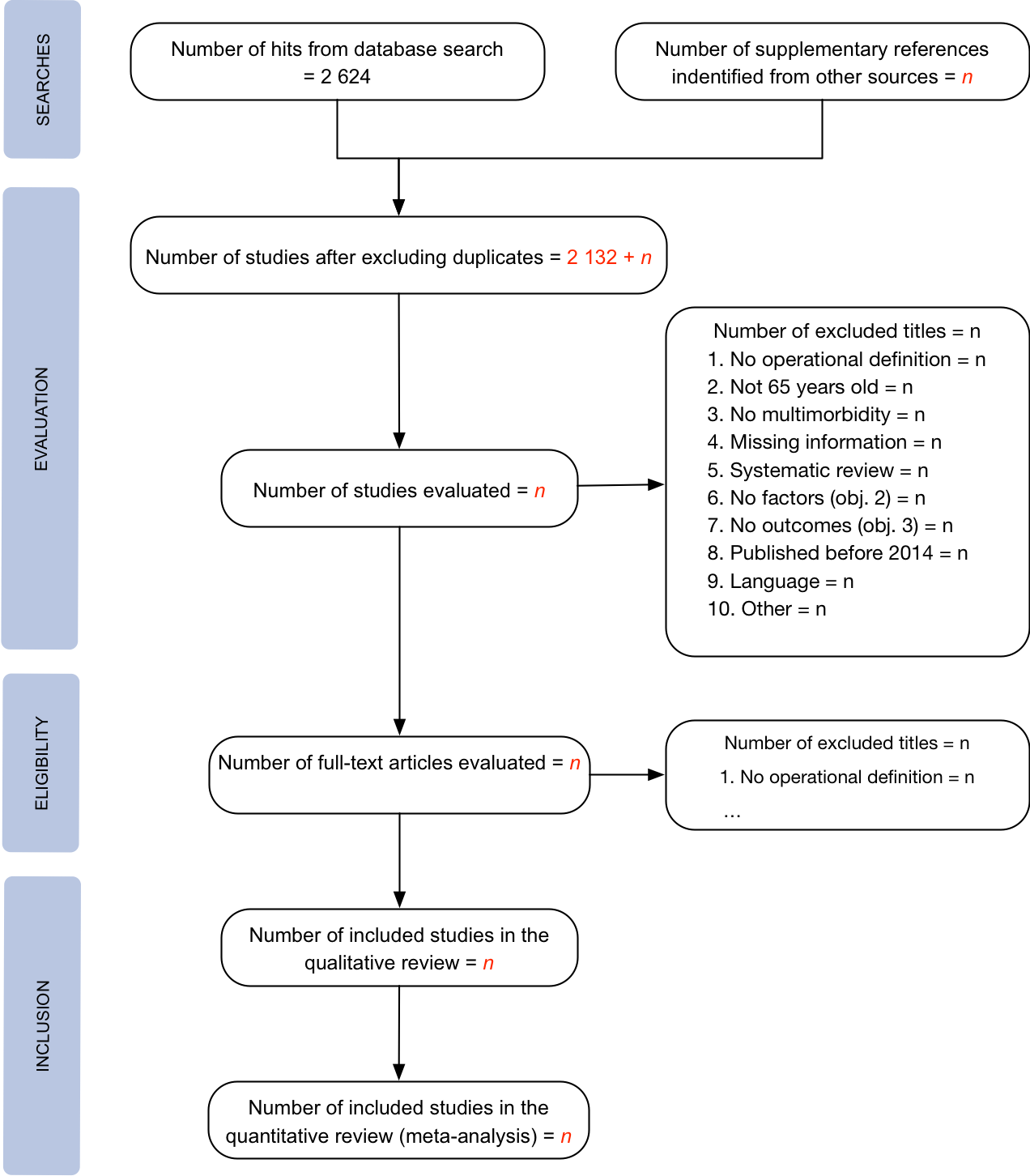

Supplement: Supplementary file 2 — PRISMA chart. (DOCX 283 kb) [file 13643_2017_492_MOESM2_ESM.docx]
